# Extendable high gain low current/high pulse modified quadratic–SEPIC converter for water treatment applications

**DOI:** 10.1038/s41598-024-55708-z

**Published:** 2024-02-28

**Authors:** P. Sumathy, J. Divya Navamani, Jagabar Sathik Mohamed Ali, A. Lavanya, Pradeep Vishnuram, Mohit Bajaj, Shir Ahmad Dost Mohammadi, Lukas Prokop

**Affiliations:** 1 Department of Electrical Engineering, SRM Institute of Science and Technology, Kattankulathur, Chennai India; 2 Department of Electrical Engineering, Graphic Era (Deemed to be University), Dehradun, 248002 India; 3 Hourani Center for Applied Scientific Research, Al-Ahliyya Amman University, Amman, Jordan; 4 Graphic Era Hill University, Dehradun, 248002 India; 5 Applied Science Research Center, Applied Science Private University, Amman, 11937 Jordan; 6 Department of Electrical and Electronics, Faculty of Engineering, Alberoni University, Kapisa, Afghanistan; 7 ENET Centre, VSB—Technical University of Ostrava, 708 00 Ostrava, Czech Republic

**Keywords:** Water treatment, High voltage pulse, High gain, Multiplier cell, HPSQB, Energy science and technology, Engineering, Mathematics and computing

## Abstract

Substantial attention has been drawn over the past few years by high step-up dc-dc converters owing to their applications in a wide range. Apart from renewable energy applications, high voltage/ high pulse converters are efficiently used in water treatment applications. The converter suggested a combination of Quadratic and SEPIC converters with a diode-capacitor cell. This topology generates high-voltage repetitive pulses with a single semiconductor switch and reduced component count. The stress across the components is less than the high-gain converters reported in the literature. The topology has an extendable feature by increasing the number of diode-capacitor cells without affecting the stress. The superiority of the high pulse generating topology is validated with a similar converter in the literature. This paper discusses the nL5 simulator results for the proposed rated topology required for water treatment. A scaled-down 50 W prototype is tested for various input voltages to generate high voltage pulse, and the analytical study is validated.

The necessity for water treatment is increasing due to various reasons like water pollution, climatic changes resulting from global warming, drinking water shortage, mainly due to the growing population, etc. Water treatment is crucial in protecting the terrestrial environment from contaminated water from industrial chemicals. The common methods for elimination of microbial contamination in water are chemical treatment^1–4^, application of intense heat^5^, filtration by UV^6,7^, electrodialysis^8^ and reverse osmosis process^9^. Conventional water treatment techniques have harmful chemical by-products during their processing. The appropriate solution to overcome the demerits of old water treatment techniques is the implementation of power electronic pulse generators, which can be used effectually for bacterial decontamination in water treatment applications. Three key water treatment techniques are involved: Electrolysis, Pulse discharging power technique and Pulse Electric field technique. The electrolysis technique used in wastewater treatment is based on the electrochemical reaction. Pulsed electric field techniques with high-voltage pulses are used to sterilize drinking water. Pulse discharge water treatment is utilized for sewage water treatment. The applications of these techniques in various water treatment sectors are illustrated in Fig. 1.

**Figure 1:**
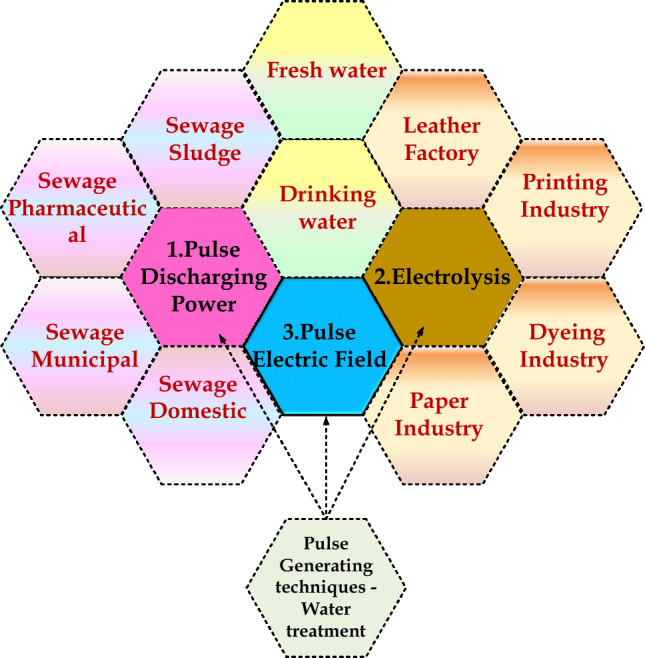
Water treatment techniques.

High gain conversion is generally achieved by increasing the multiplier cell stages. Topologies discussed in the literature include a modular bipolar high-voltage pulse generator capable of generating bipolar pulses with high voltage and output flexibility^10–13^. A two-stage high-voltage pulse generator converter topology with key features like scalability, modularity, and redundancy is used for electroporation applications is discussed^14^. Sequential pulse generators producing repetitive pulses were discussed as suitable for disinfection applications^15^. Pulsed Electric Field (PEF) method has had promising applications in several fields in the last few years. Pathogenic bacteria and other antibiotic–resistant microorganisms are treated with the high pulse of the electric field. For water treatment applications, pulsed arc discharge and underwater pulsed streamer corona discharge are the two main types of Pulsed Electric Field (PEF) treatments^16,17^. The effect of microorganisms and their removal by comparing the two methods is presented in^17^. It is also observed from the literature that the underwater pulsed streamer corona discharge requires lesser power compared to pulsed arc discharge. The application of bipolar pulses is even more effective compared to its counterpart. Even though electroporation successfully applies pulsed electric fields, permanent cell membrane damage is caused by the electric voltage of very high^18–20^. Generally, the pulse generation for water treatment based on power electronic switches can be classified into two types precisely classical and solid-state pule generation methodology. Different pulse generation methodologies are depicted in Fig. 2. The Chopper and Marx circuits generate pulses with capacitor storage in classical methods. In Magnetic Pulse Compressor (MPC), a storage capacitor and a magnetic switch is employed for pulse generation. PFL is a crucial method to generate high-power short pulses of the nanoseconds pulse width. For generating rectangular pulses with a pulse width greater than 500 ns, PFL is unsuitable; therefore, Pulse Forming Network (PFN) is used. Small and medium power pulses are generated in a dual resonant Tesla transformer circuit.

**Figure 2:**
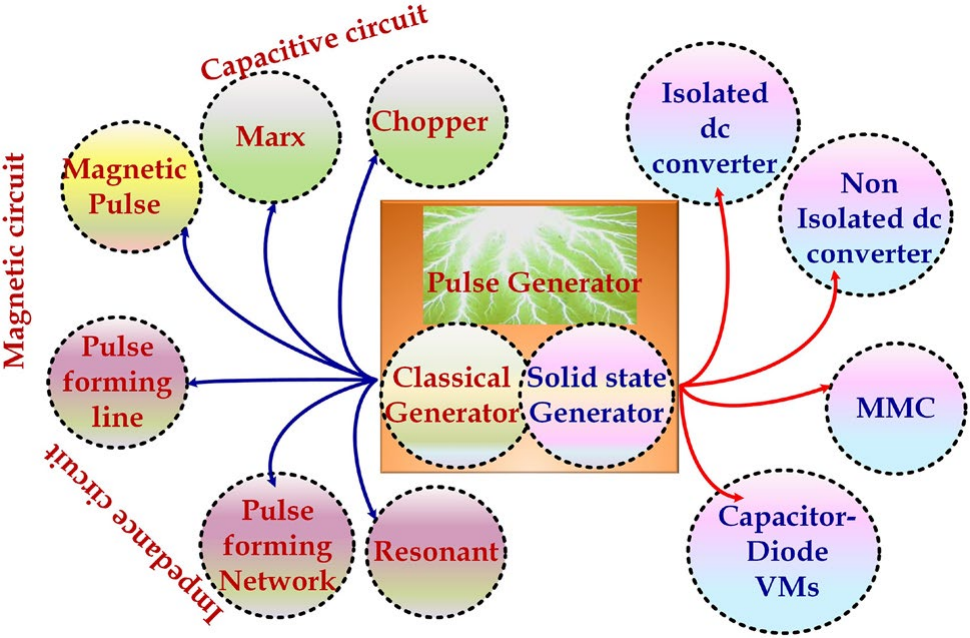
Pulse generation methodologies.

In solid-state pulse generation methodology, solid-state switches generate definite pulses. DC-DC isolated and non-isolated converters generate high-voltage pulses with single or multiple controls. This methodology can be used wherever high-voltage pulses are required in water treatment applications. Capacitor–diode voltage multipliers (CDVMs) are also used in electric pulse generators which are highly reliable, efficient, lightweight, and smaller in size^19^. However, they pose a risk of increasing voltage ripples of capacitors and falling output pulse frequency. Low pulsed dc voltages effectively deactivating microbes generally found diffused in water from dead animals is discussed in^21–23^. Taphylococcus aureus is deactivated with 50–80 V pulses for 5 min for 3 days^21^ followed by pichia rhodanensis with 400–500 V pulses^22^ and pseudomonas aeruginosa with 500 V pulses for 100 μs^23^.

An adjustable pulse magnitude, pulse width and pulse count electric pulse generator are validated with PSIM simulation for 1.5 kV to produce unipolar and bipolar pulses leading to longevity and reliability^24^. Apart from voltage source topology, a current source topology is made with a series-connected Bi-MOSFET switch. A repetitive pulse or single pulse is produced as desired and the current controls the voltage magnitudes of the output pulses through the inductor in every discharge cycle of the load^25^. A state-space model is derived for another continuous inductor current operation mode and results are validated with a 50W prototype^26^. A 10 kV and 1 kW prototype experimental results were presented for a solid-state pulse power modulator for producing short pulses with high switching speed using a new gate driving circuit^27^. The role of the pulsed electric field in the water treatment process is illustrated with a flow diagram in Fig. 3. Based on the challenges addressed in the literature^28–36^, the following topology is proposed in this paper.

**Figure 3:**
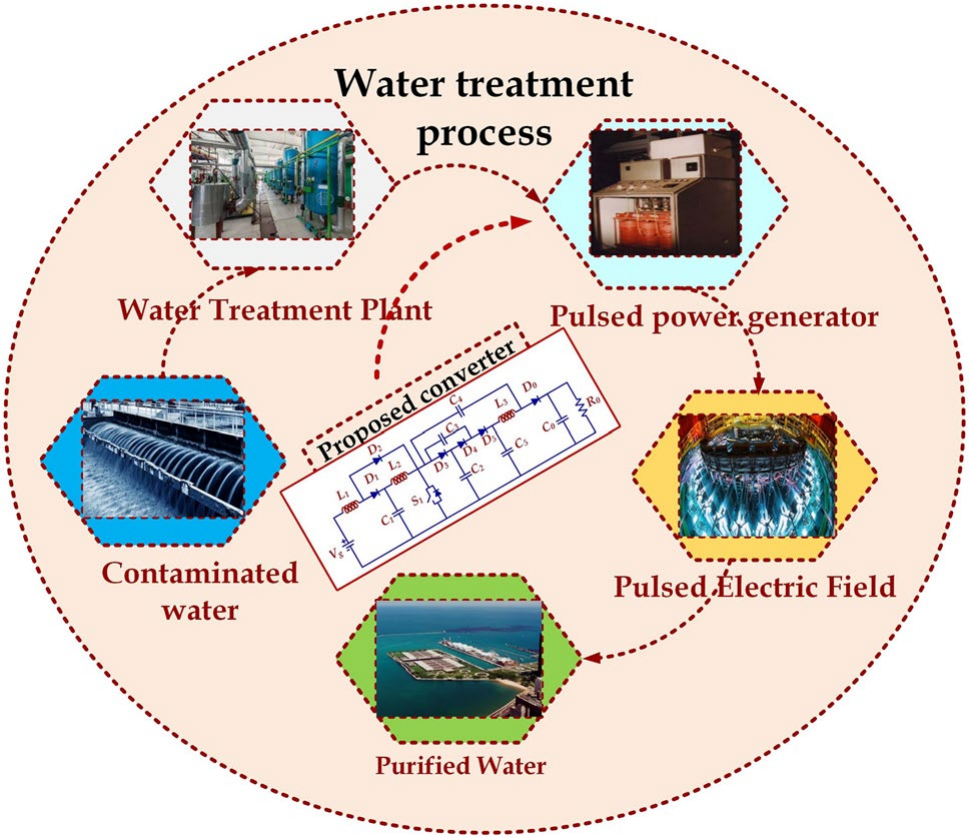
Block diagram of water treatment application.

In this paper, a novel high-voltage pulse generator based on Quadratic and SEPIC dc converters in combination with multiple diode-capacitor cell is proposed. The derived high pulse generator affords the following features:

(i) Single switch topology
(ii) Extendable feature
(iii) Lesser component count.

Despite these advantages, the suggested high-pulse generator requires a high-voltage switch to chop the continuous dc voltage to a high-voltage pulse. However, this issue can be overcome by using a series-connected switch. The major contributions of the article are.

- Proposed novel single switch topology with extendable features.
- Reliability analysis of proposed topology is performed by considering annual mission profile of the water treatment plant and compared with similar topology in the literature.
- With the process parameters, water sample is tested before and after treatment and the presence of microbes are analyzed.
- Sample is tested with 360 V and 5000 V to observe the significance of the magnitude of pulsed voltage.

This paper is organized as follows: Following the introduction, a description of the proposed topology is presented in Section "Proposed repetitive high pulse generating topology". The design of the derived topology with the voltage stress across the components is illustrated in Section "Design and component analysis". The superiority of the suggested converter is highlighted by comparing it with a similar topology in the literature in Section "Performance analysis". Simulation results are presented in Section "Comparative Study" by using nL5 software. The scaled-down prototype is tested and the results are depicted in Section "Simulation results". Finally, the article is concluded in the last section.

## Proposed repetitive high pulse generating topology

This section performs the derivation, operating principle, and steady-state analysis of the topology. The operating principle is discussed with a single diode-capacitor cell for simplicity.

### High pulse modified SEPIC-quadratic boost DC converter

**Figure 4:**
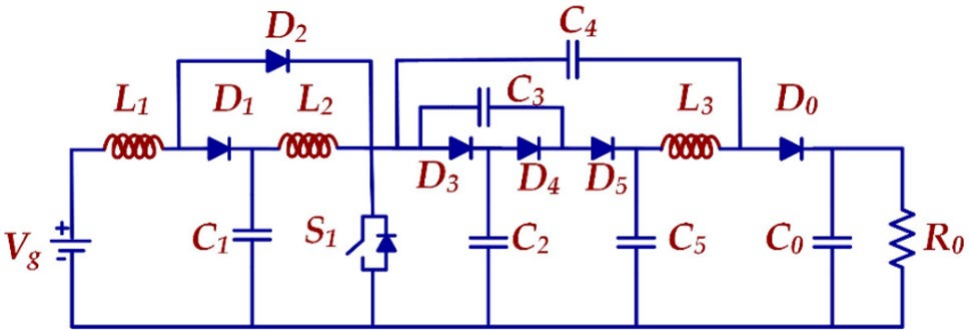
High pulse modified Sepic- Quadratic Boost dc-dc converter.

The schematic circuit of the HPSQB converter is presented in Fig. 4. The converter's gain increases by proportionately increasing the voltage multiplier stages.

### Operating principle

Based on the conducting and non-conducting conditions of the semiconductor switch, the working of the converter is divided into two modes.

**Figure 5:**
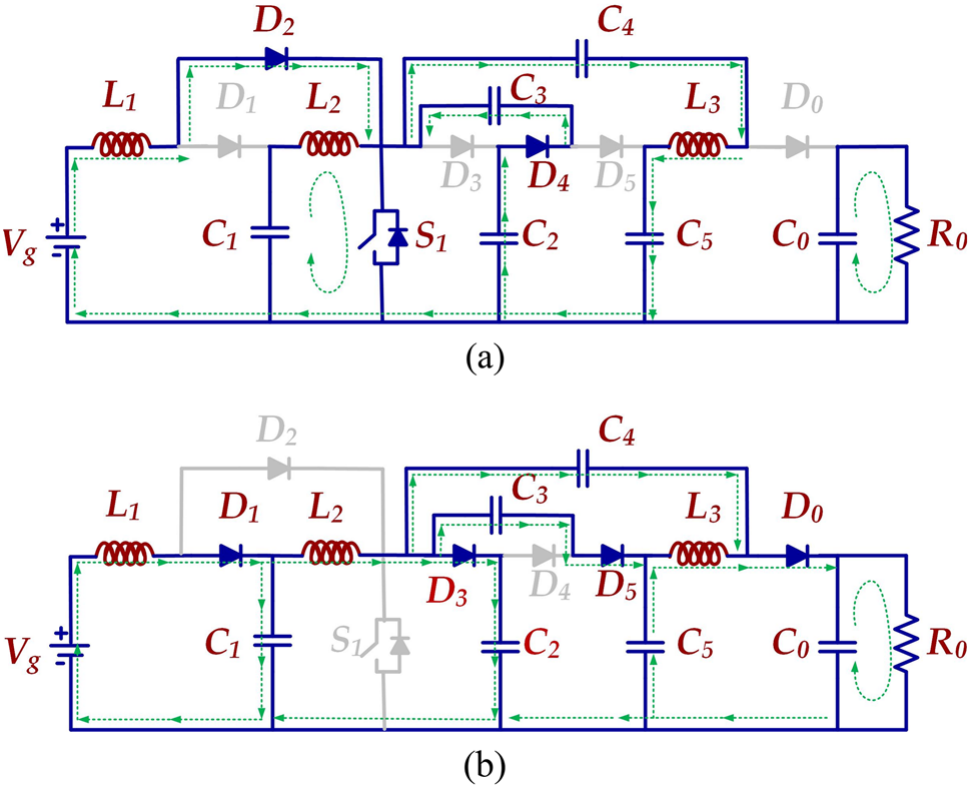
Operating principle (**a**) Mode-I (**b**) Mode-II.

**Figure 6:**
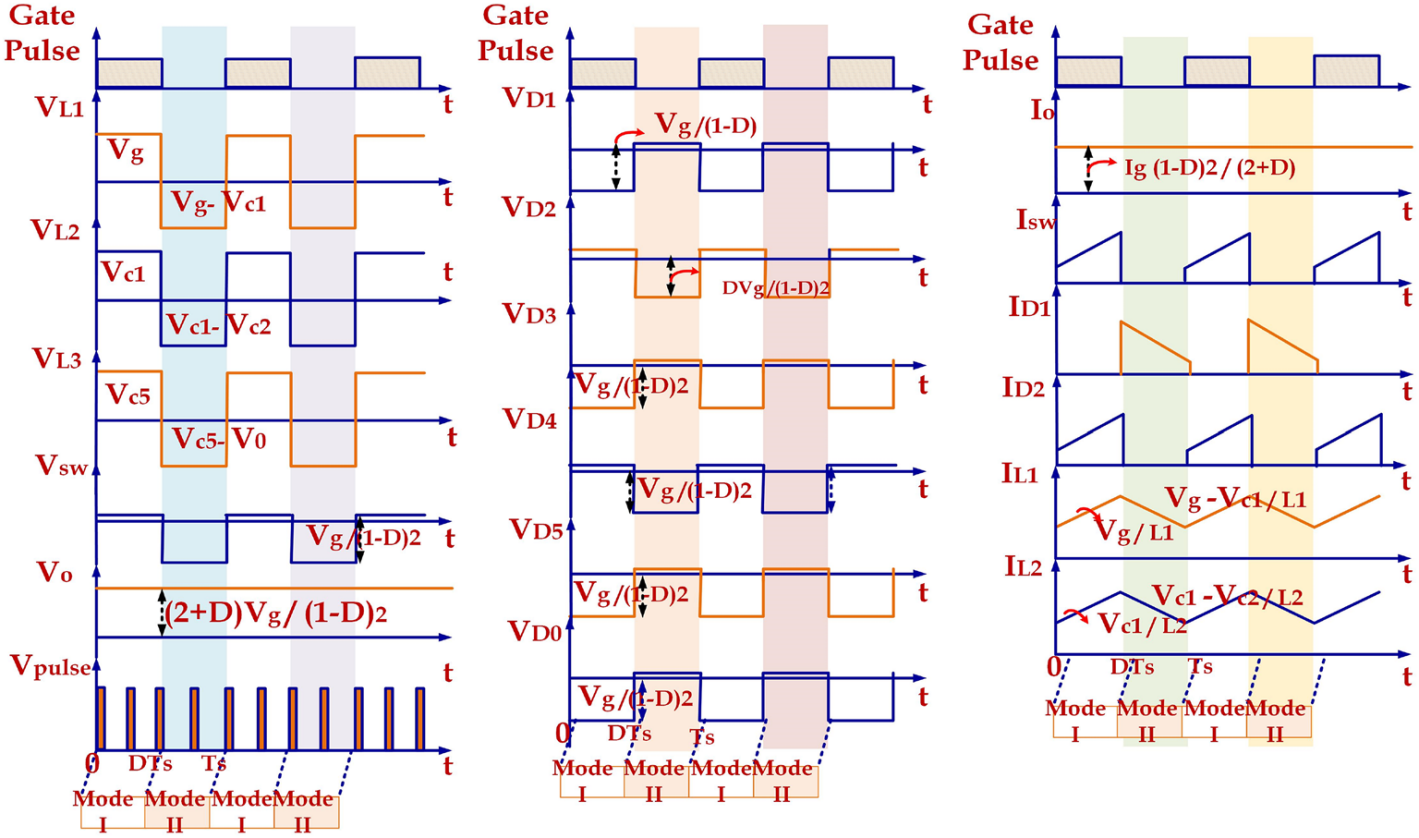
Key waveforms of the proposed topology.

*Mode-I*: Fig. 5a depicts the equivalent circuit of the proposed topology in the ON mode of the switch. In Fig. 5a, the Mode-I is from 0 to DTs, representing the duty cycle as ‘D’ and the switching period as Ts. The switch is in ‘ON’ condition in this mode. During this mode, the input supply, Vg, charges the inductor ‘L1’ and forms a closed circuit with diode D2 while diode D1 is not conducting. Inductor L_2_ is charged from the capacitor C_1_. The non-conducting state of the diodes D_3_ and D_5_ and conducting state of diode D_4_ connects the capacitors C_2_ and C_3_ in series and capacitors C_4_ and C_5_ in series. The series combination of C_2_, C_3_ and C_4_, C_5_ branches falls in parallel with each other and forms a closed circuit. The load at this condition is fed from the output capacitor ‘C_O_’. Diodes D_1_, D_3_, D_5,_ and D_O_ are in a non-conducting state during this mode. The key waveforms of the HPSQB converter are also depicted in Fig. 6. V_pulse_ is the voltage obtained after the high voltage switch which is used to chop the output voltage of the converter.

*Mode-II*: As given in Fig. 5b. The Mode-II is from DTs to Ts. In this mode, the inductors L_1_ and L_2_ discharges to the load along with the input supply Vg. Diodes D_2_ and D_4_ are in blocking state and diodes D_2_, D_3_, D_5_ and D_O_ moves into conduction state. The output voltage in this mode is equal to V_C5_ + V_C4_–V_C3_.

### Steady-state analysis in CCM

The voltage gain of the proposed converter is derived mathematically by applying the magnetic flux principle on charging and discharging state of the inductor. Considering the internal resistance of the proposed converter as zero or negligible. Table 1 presents the voltage expression of the inductor in ON and OFF modes. The derivation of the voltage gain is derived as follows:

**Table 1:** Voltage expression of the inductor.

ON state	OFF state
${V}_{L1}={V}_{Cg}$	${V}_{L1}={V}_{g}-{V}_{C1}$
${V}_{L2}={V}_{C1}$	${V}_{L2}={V}_{C1}-{V}_{C2}$
${V}_{L3}={V}_{C5}-{V}_{C4}$	${V}_{L3}={V}_{C5}-{V}_{O}$

On application of the volt-sec balance principle, the voltage gain of the proposed converter is thus derived as,

1 $${G}_{V}=\frac{{V}_{O}}{{V}_{g}}=\frac{(2+D)}{{\left[1-D\right]}^{2}}$$

The generalized expression of the converter proposed with ‘M’ number of multiplier stages is given as,

2 $${G}_{V}=\frac{{V}_{O}}{{V}_{g}}=\frac{M(2+D)}{{\left[1-D\right]}^{2}}$$

The current gain of the converter proposed is obtained as,

3 $$\frac{{I}_{O}}{{I}_{g}}=\frac{{\left[1-D\right]}^{2}}{(2+D)}$$

**Figure 7:**
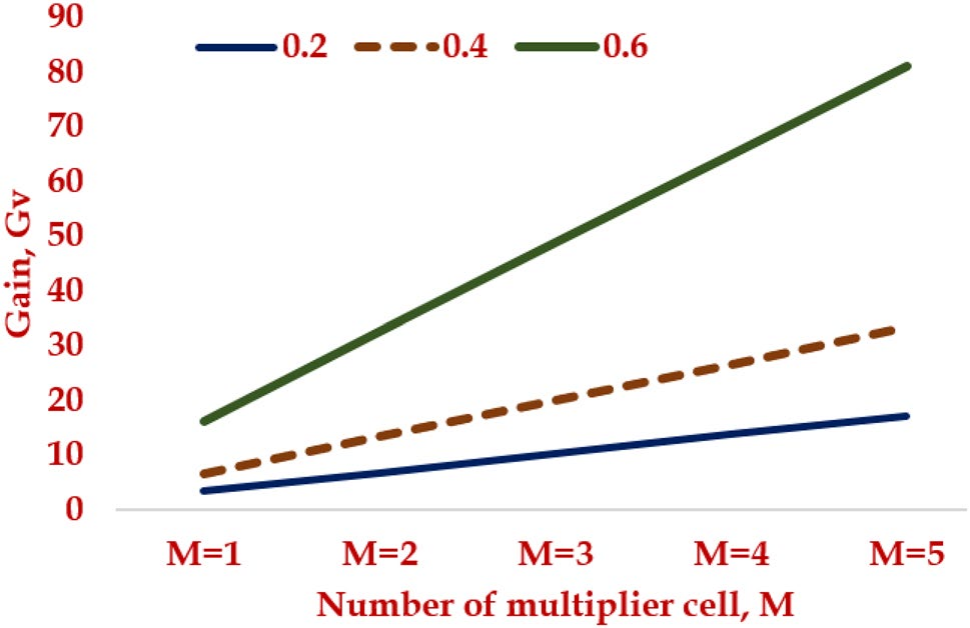
Gain of the HPSQB topology with increase in multiplier cell, M.

The variation of voltage gain, Gv with the multiplier cell, M is presented in Fig. 7 for M = 1 to 5.

## Design and component analysis

This section elaborates on the steps to design the proposed high-pulse generator. Since the required pulse for the water treatment application is observed to be in kV. Accordingly, the passive components of the converter are acquired. The stress across the topology components is also analyzed and presented to highlight the advantage of the derived topology.

### Design of passive components

The design expression of the inductors (L_1_, L_2_ and L_3_) in the proposed topology (number of multiplier cell, M = 1) is

4 $${L}_{1}=\frac{{R}_{O}(1-D{)}^{4}D}{2(2+D{)}^{2}{f}_{s}}$$

5 $${L}_{2}=\frac{{R}_{O}(1-D{)}^{2}D}{2(2+D{)}^{2}{f}_{s}}$$

6 $${L}_{3}=\frac{{R}_{O}(1-D)D}{2(2+D){f}_{s}}$$

The capacitor (C_1_, C_2_, C_3_, C_4_, C_5_ and C_O_) formula

7 $${C}_{1}=\frac{(2+D)D{I}_{O}}{{f}_{s}(1-D)\Delta [{V}_{g}/(1-D)]}$$

8 $${C}_{2}={C}_{3}=\frac{{I}_{O}(1-D)}{{f}_{s}\Delta [Vg/(1-D{)}^{2}]}$$

9 $${C}_{4}=\frac{{I}_{O}D}{{f}_{s}\Delta [Vg(1+D)/(1-D{)}^{2}]}$$

10 $${C}_{5}=\frac{{I}_{O}D}{{f}_{s}\Delta [2Vg/(1-D{)}^{2}]}$$

11 $${C}_{O}=\frac{{I}_{O}}{{f}_{s}\Delta [(2+D)Vg/(1-D{)}^{2}]}$$

### Stress across the components

The voltage and current stress of the passive and semiconductor components are derived from the steady-state analysis for designing the converter. Table 2 illustrates the voltage stress across the semiconductor devices for a different number of multiplier cells, M. From this Table, it is observed that the stress across the components is independent of the number of multiplier cells. The increase in the M number of multiplier cells does not affect the stress value.

**Table 2:** Voltage expression of the inductor.

Number of multiplier cell	Voltage gain	Voltage stress on switch in terms of V_g_	Voltage stress on Diode D1	Voltage stress on Diode D2	Voltage stress on Diode D3 and D4	Voltage stress on Diode D5	Voltage stress on Diode Do
1	$\frac{(2 + D)}{{[1 - D]^{2} }}$	$\frac{{V_{g} }}{{[1 - D]^{2} }}$	$\frac{{V_{g} }}{[1 - D]}$	$\frac{{V_{g} D}}{{[1 - D]^{2} }}$	$\frac{{V_{g} }}{{[1 - D]^{2} }}$	$\frac{{V_{g} }}{{[1 - D]^{2} }}$	$\frac{{V_{g} }}{{[1 - D]^{2} }}$
2	$\frac{2(2 + D)}{{[1 - D]^{2} }}$						
3	$\frac{3(2 + D)}{{[1 - D]^{2} }}$						
n	$\frac{M(2 + D)}{{[1 - D]^{2} }}$						

### Stress across the capacitors

Pulsed electric field application mainly depends on charging and discharging the capacitors in the converter. The voltage stress across the capacitors C_1_, C_2_, C_3_, C_4_, C_5_ and C_O_ is

12 $${V}_{C1}=\frac{{V}_{g}}{\left[1-D\right]}$$

13 $${V}_{C2}={V}_{C3}=\frac{{V}_{g}}{{\left[1-D\right]}^{2}}$$

14 $${V}_{C4}=\frac{{V}_{g}(1+D)}{{\left[1-D\right]}^{2}}$$

15 $${V}_{C5}=\frac{2{V}_{g}}{{\left[1-D\right]}^{2}}$$

### Current stress of the components

The RMS current through the inductor is given as

16 $${I}_{L1}=\frac{{I}_{O}(2+D)}{{\left[1-D\right]}^{2}}$$

17 $${I}_{L2}=\frac{{I}_{O}(2+D)}{1-D}$$

18 $${I}_{LO}={I}_{O}$$

The RMS current through the switch, S_1_ is given as

19 $${I}_{S1}=\frac{{I}_{O}\left[4-{D}^{2}\right]\sqrt{D}}{{\left[1-D\right]}^{2}}$$

## Performance analysis

### Efficiency analysis

**Figure 8:**
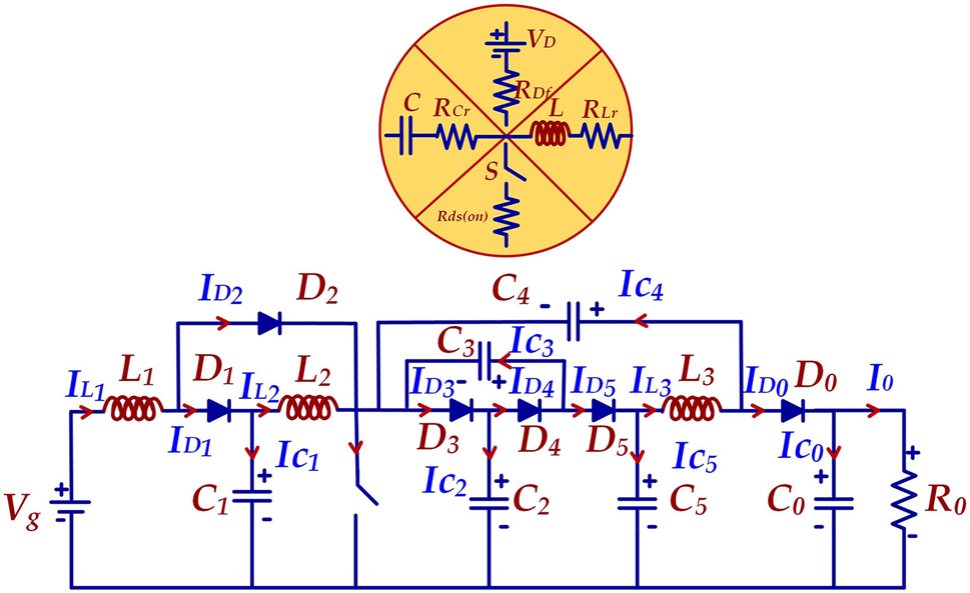
Equivalent circuit of the HPSQB topology.

Figure 8 presents the equivalent circuit of the HPSQB converter for efficiency analysis. For efficiency calculation of the proposed converter, the parasitic resistance of components is considered as shown in the equivalent circuit and the equations as follows:

20 $${P}_{s}={{I}_{s(rms)}}^{2}{R}_{ds(on)}$$

21 $${P}_{D1}=[{V}_{F}({i}_{D1(avg)}+{i}_{D2(avg)}+{i}_{D3(avg)}+{i}_{D4(avg)}+{i}_{D5(avg)})+{V}_{F}{i}_{D0(avg)}]$$

22 $${P}_{D2}={R}_{f}[{{i}_{D1(rms)}}^{2}+{{i}_{D2(rms)}}^{2}+{{i}_{D3(rms)}}^{2}+{{i}_{D4(rms)}}^{2}+{{i}_{D5(rms)}}^{2}]+{R}_{F}{{i}_{D0(rms)}}^{2}]$$

23 $${P}_{D}={P}_{D1}+{P}_{D2}$$

24 $${P}_{L}={{I}_{L1(rms)}}^{2}{r}_{L1}+{{I}_{L2(rms)}}^{2}{r}_{L2}+{{I}_{L3(rms)}}^{2}{r}_{L3}$$

25 $${P}_{C}={{I}_{C1(rms)}}^{2}{r}_{C1}+{{I}_{C2(rms)}}^{2}{r}_{C2}+{{I}_{C3(rms)}}^{2}{r}_{C3}+{{I}_{C4(rms)}}^{2}{r}_{C4}+{{I}_{C5(rms)}}^{2}{r}_{C5}+{{I}_{C0(rms)}}^{2}{r}_{C0}$$

The power rating of HPSQB converter is considered as 500 W. The input and output voltage are chosen as 300 V and 35.1 kV respectively as mentioned in simulation section.

Table 3 presents the specification considered for the efficiency study of the HPSQB converter. With (20–25), the losses across the converter components are obtained and the efficiency analysis is performed. Efficiency analysis is performed for the specification, as shown in Table 3, similar to the simulation study. The average and RMS current few diodes in the topology are given in Table 4, along with the losses in the components. Similarly, the capacitor loss is also performed and the total loss is observed to be 67 W. With this loss, the efficiency of the HPSQB topology is calculated as 87%.

**Table 3:** Specification considered for efficiency analysis.

Parameters	Rating	Type/model
Power	500 W	–
Duty cycle	0.61	
Switching frequency	50 kHz	
Switch	2 kV/5 A Rds(on) = 4.2 Ω	IXTF6N200P3

Diode	3.3 kV/10 A V_f_ = 1.5 V/R_f_ = 0.3 Ω	GB05MPS33-263

Inductor	10 mH R_L_ = 165 mH	
Capacitor	1 µF/ 2.5 kV

**Table 4:** Losses across the components.

Components	Expression	Loss	Total loss
Switch	${I}_{S1(rms)}=\frac{{I}_{O}\left[4-{D}^{2}\right]\sqrt{D}}{{\left[1-D\right]}^{2}}$	P_sw(cond)_ = 46 W	59 W
		P_sw(switching)_ = 13 W	
Diode	${I}_{D1(avg)}=\frac{{I}_{O}(2+D)}{1-D}$	P_D1_ = 1.29 W	3.6 W
		P_D2_ = 2.02 W	
	${I}_{D2(avg)}=\frac{{I}_{O}(2+D)D}{{\left[1-D\right]}^{2}}$	⋮	
	${I}_{D1(rms)}=\frac{{I}_{O}(2+D)(\sqrt{(1-D)}}{{\left[1-D\right]}^{2}}$	P_D0_ = 0.1 W	
	$${I}_{D2(rms)}=\frac{{I}_{O}(2+D)(\sqrt{D}}{{\left[1-D\right]}^{2}}$$		
Inductor	${I}_{L1}=\frac{{I}_{O}(2+D)}{{\left[1-D\right]}^{2}}$	P_L1_ = 3.23 W	3.3 W
		P_L2_ = 0.069 W	
	${I}_{L2}=\frac{{I}_{O}(2+D)}{1-D}$	P_L3_ = 0.01 W	
	$${I}_{LO}={I}_{O}$$		

### Sensitivity analysis

Sensitivity analysis is recommended in designing a novel converter because it portrays the sensitivity of voltage gain for the change in D, duty cycle, and RL, the internal resistance of the inductor. The efficiency of the topology is

26 $$\eta ={G}_{V}{G}_{I}$$

27 $${G}_{V}=\frac{\eta (2+D)}{(1-D{)}^{2}}$$

The voltage conversion ratio of the HPSQB topology with the R_L_ of inductor is

28 $${G}_{V}=\frac{(2+D)}{(1-D{)}^{2}\left[1+\frac{(2+D{)}^{2}{R}_{L1}}{{R}_{O}(1-D{)}^{4}}+\frac{(2+D{)}^{2}{R}_{L2}}{{R}_{O}(1-D{)}^{2}}+\frac{{R}_{L3}}{{R}_{O}}\right]}$$

The voltage conversion ratio is simplified by considering same internal resistance for all the inductor (R_L1_ = R_L2_ = R_L3_)

29 $${G}_{V}=\frac{{R}_{O}(2+D)(1-D{)}^{2}}{{R}_{O}(1-D{)}^{4}+(5-2D{)}^{2}{R}_{L}}$$

Sensitivity analysis of voltage gain is accomplished by differentiating (29) with respect to D. After mathematical manipulation, the final yielded equation is

30 $$\frac{d{G}_{V}}{dD}=\frac{\left[{R}_{O}(1-D{)}^{4}+(5-2D{)}^{2}{R}_{L}\right][3{D}^{2}-3]+[{R}_{O}(2+D)(1-D{)}^{2}][4{R}_{O}(1-D{)}^{3}+(10-4D){R}_{L}]}{{\left[{R}_{O}(1-D{)}^{4}+(5-2D{)}^{2}{R}_{L}\right]}^{2}}$$

Similarly, the sensitivity of the output voltage with respect to D is

31 $$\frac{d{V}_{O}}{dD}=\frac{3[{D}^{2}-1]}{(1-D{)}^{4}}$$

**Figure 9:**
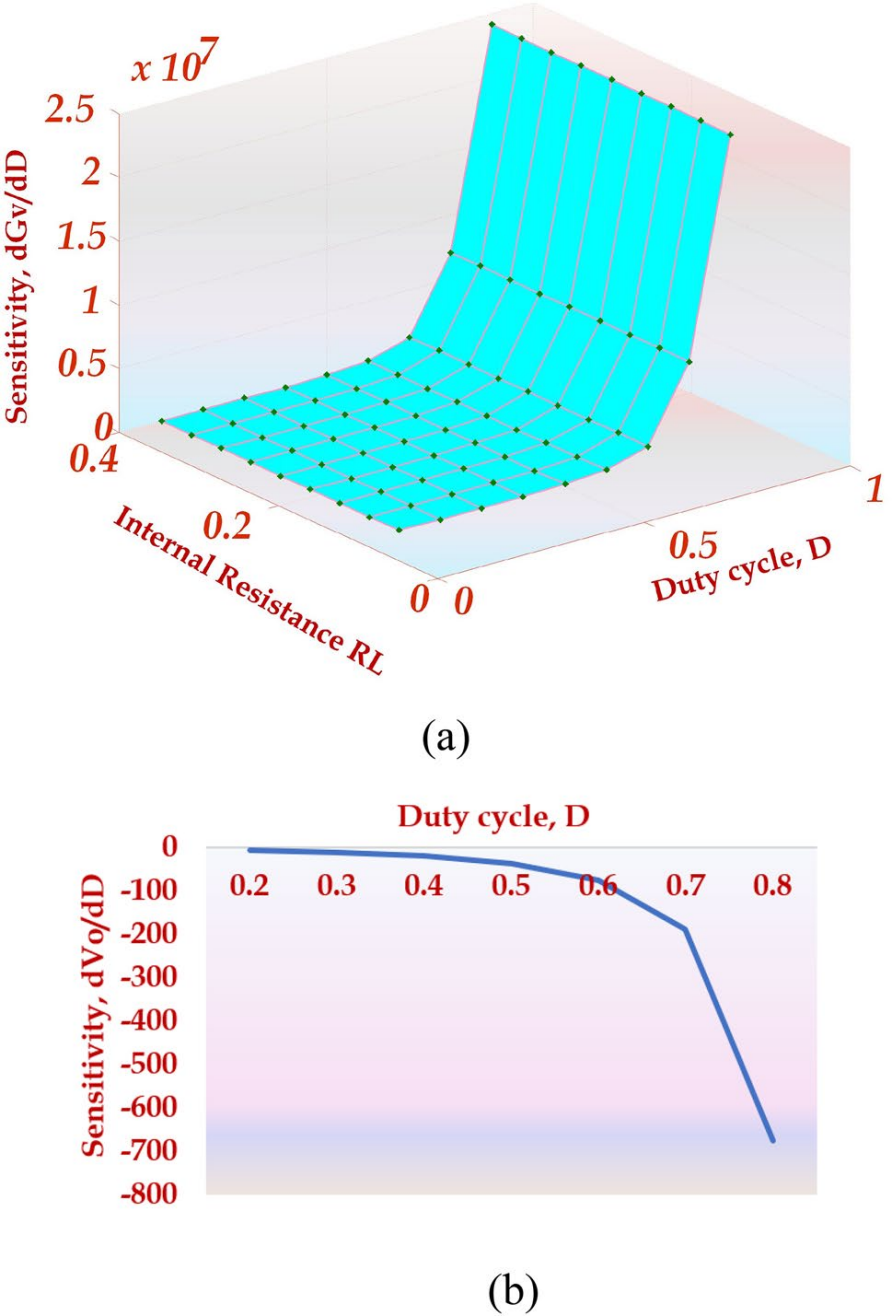
Sensitivity analysis of HPSQB topology (**a**) dGv/dD (**b**) dVo/dD.

The sensitivity analysis of voltage gain and output voltage for duty cycle D is performed and depicted in Fig. 9a,b. It is depicted in Fig. 9a that the variation of the non-ideal voltage gain of the HPSQB converter for a higher duty cycle is significant for the internal resistance of the inductor, RL. Similarly, the variation of ideal voltage gain for duty cycle D is predominant for the duty cycle greater than 0.5, which is illustrated in Fig. 9b.

### Reliability analysis

**Figure 10:**
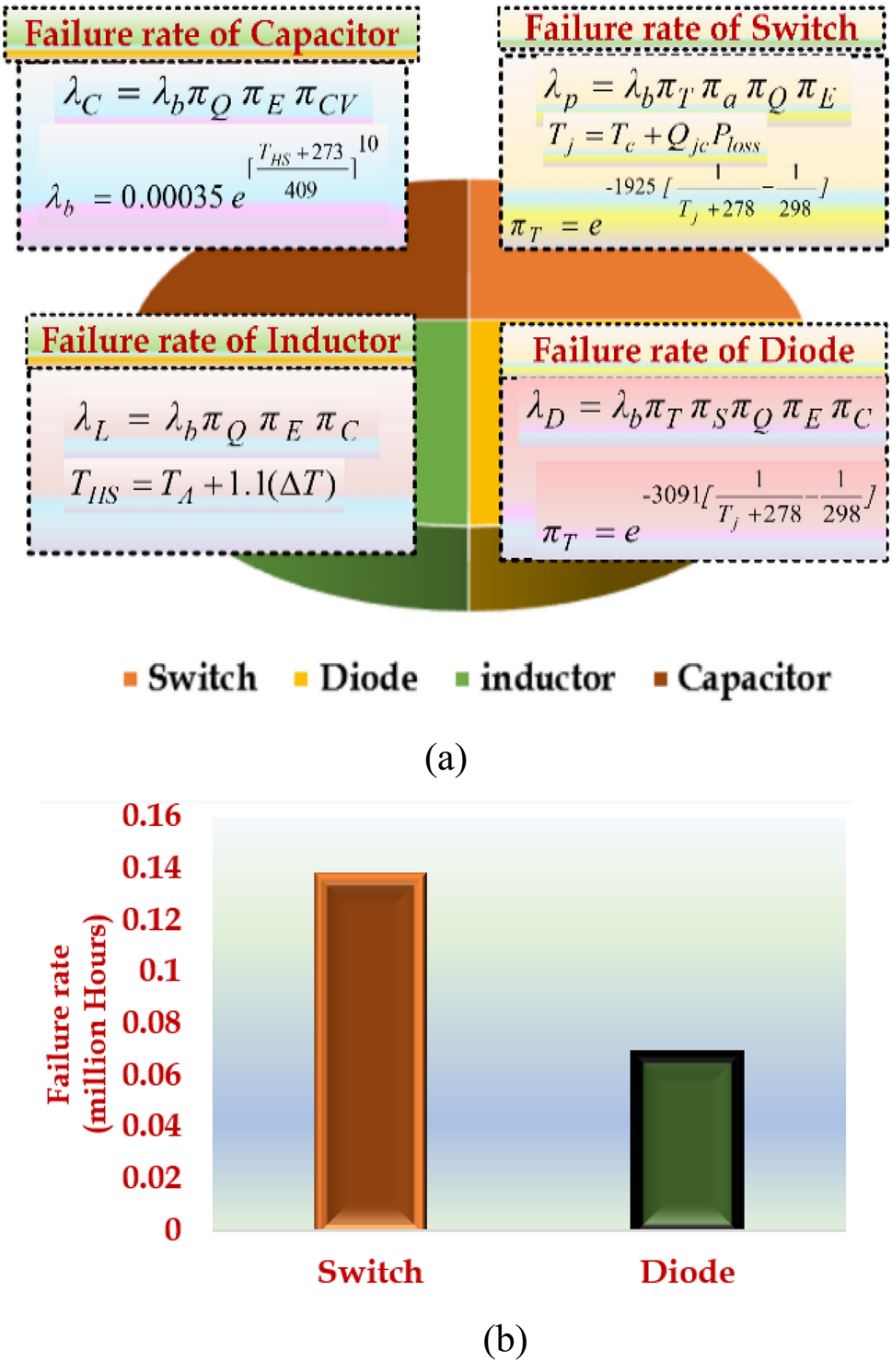
Parameters related to failure rate calculation.

This section determines the failure rate based on the equations given in Fig. 10a. The military handbook calcifies the failure rate in many applications to determine reliability. Accurate reliability predictions are essential in applications that depend on various parameters, such as the physical and operating characteristics of the converter and its environment. The reliability analysis of HPSQB topology determined that the semiconductor devices are failure prone compared to the passive components. Therefore, the failure rate of the switch and diodes are discussed and depicted in Fig. 10b. The failure rate of the switch is estimated to be greater than the diodes in the converter circuit considered.

The reliability analysis is extended by considering the annual mission profile of the water treatment plant in Chennai, Tamil Nadu and it is presented in Table 5. This analysis is validated by comparing the MTTF computation of Proposed HPSQB converter with the pulsed voltage converter proposed in^19^. Table 6 depicts the comparison performed in the MTTF calculation of HPSQB converter with the converter in^19^. From this comparative study, it is observed that HPSQB converter’s reliability is better compared to the topology presented in^19^.

**Table 5:** Annual mission profile of water treatment plant.

Condition	Time (hrs)	On/Off	Ambient Temperature (°C)	Relative Humidity %	∆T (°C)	Number of Cycles per year	Duration of Cycles (hrs)	Maximum temperature during cycling (°C)
Day/On	3600	On	40	50	15	300	12	45
Day/Off	780	Off	35	35	10	65	12	40
Night/Off	4380	Off	30	40	5	365	12	35

**Table 6:** MTTF computation.

Component failure rate	Proposed HPSQB converter	Converter in Ref^19^
λ_S_	2.24 × 10^−6^	2.32 × 10^−6^
λ_D_	3.49 × 10^−6^	5.43 × 10^−6^
λ_C_	0.0002 × 10^−6^	0.009 × 10^−6^
λ_L_	0.82 × 10^−6^	0.022 × 10^−6^
Mean time to failure (MTTF)	17.44Years	14.67Years

## Comparative Study

**Figure 11:**
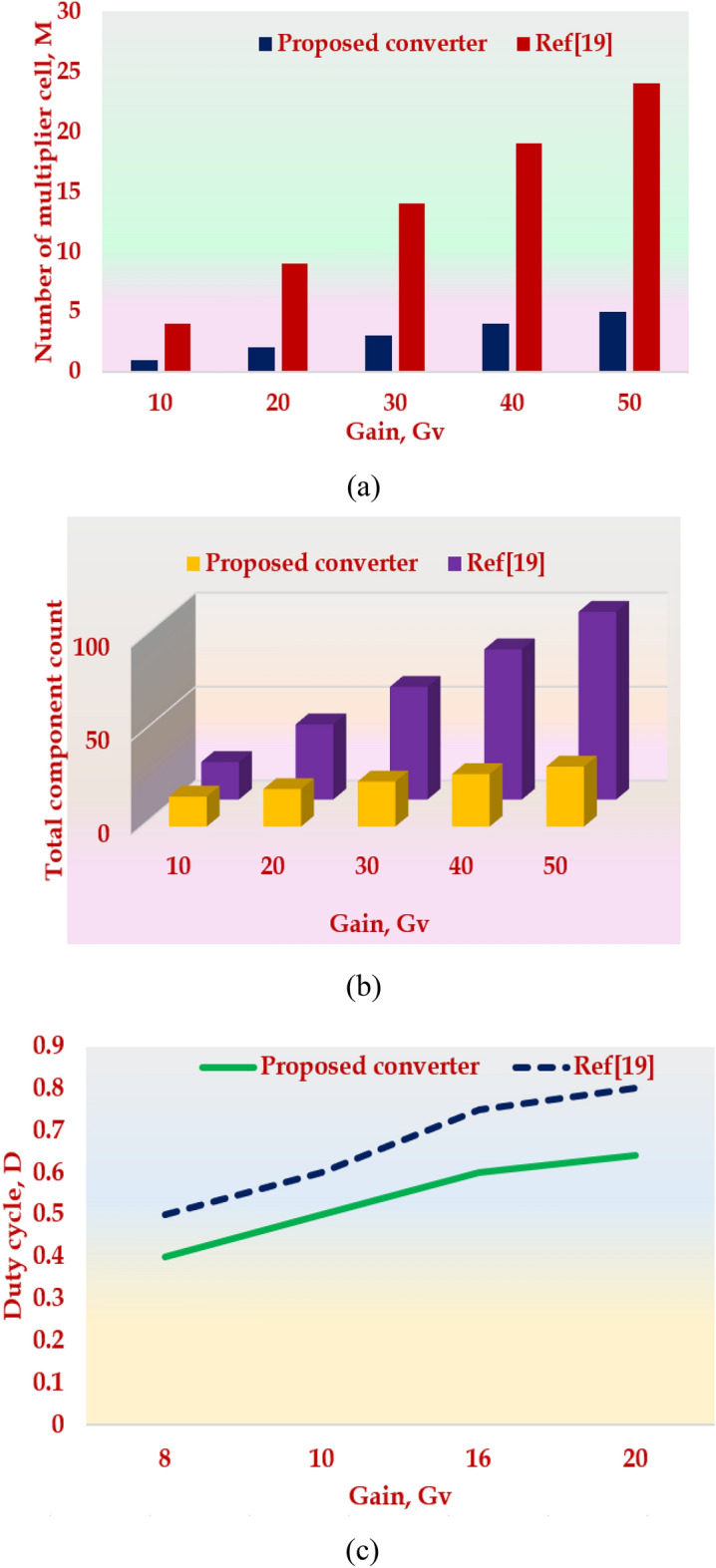

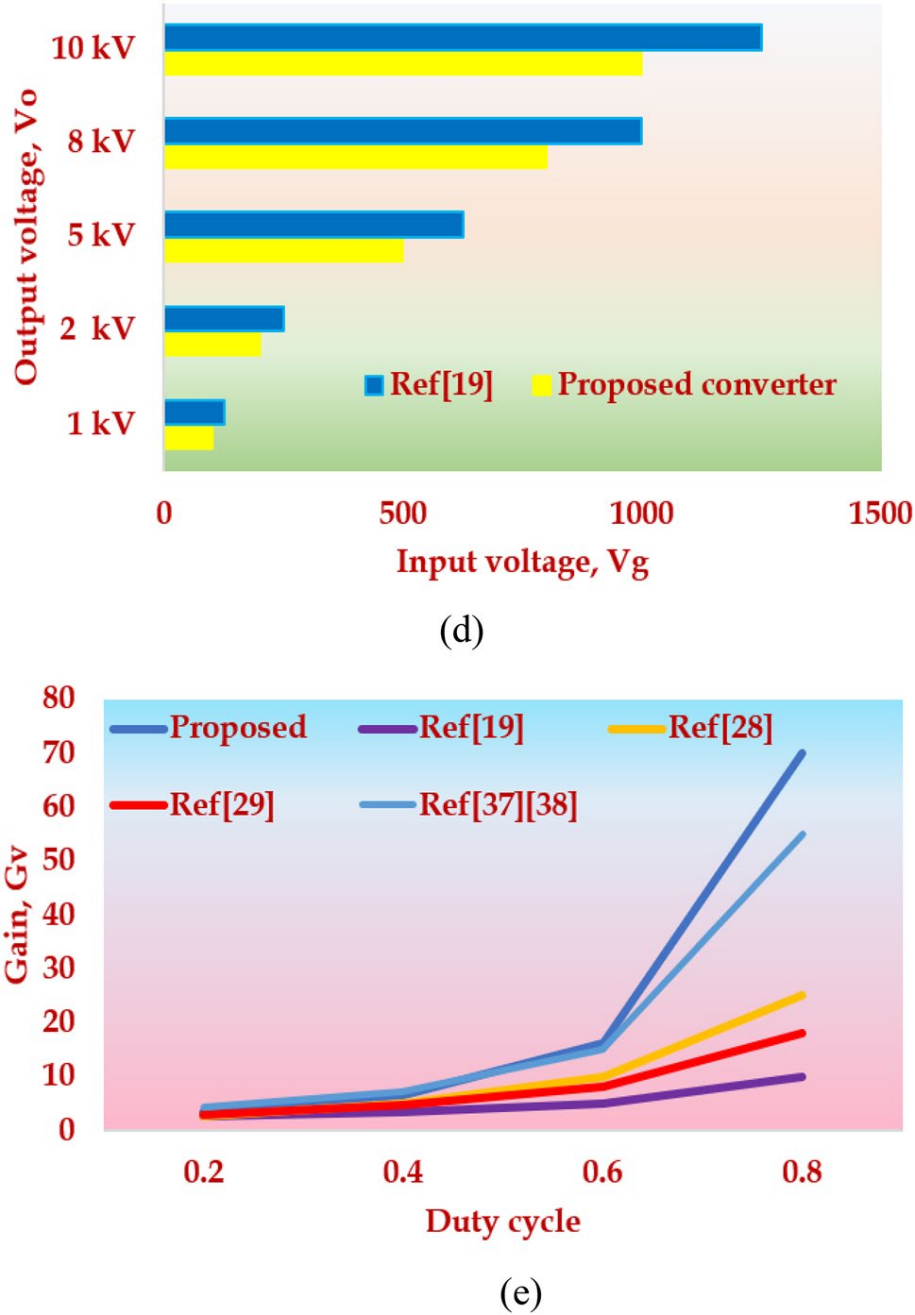
Key highlights of the HPSQB topology (**a**) Gain versus voltage multiplier cell (**b**) Gain versus total component count (**c**) Gain versus duty cycle (**d**) Input versus output voltage (**e**) Gain comparison with similar topologies in literature.

This section compares the topology with similar topologies in the literature and proves its superiority. First, the derived topology is compared with the voltage multiplier-based topology suggested for water treatment applications^19^. Figure 11a–d presents the comparison made on the derived topology with the topology in^19^. In Fig. 11a,b, the duty cycle is kept constant at 0.5 and the number of multiplier cells, M and the total component count is analyzed for various values of Gain, Gv. The comparative Table 7 shows that the proposed converter uses lesser components to achieve the same voltage gain as compared to the converter in^19^. In Fig. 11c, the total component count is kept constant at 16 and the duty cycle is compared for various values of Gain, Gv.

**Table 7:** Comparative study of the HPSQB topology with ref^19^.

Parameters	Topology	Duty cycle, D = 0.5				
		Gain, Gv				
		10	20	30	40	50
Number of Multiplier cell, M	Proposed converter	1	2	3	4	5
	Ref^19^	4	9	14	19	24
Total component count	Proposed converter	16	20	24	28	32
	Ref^19^	20	40	60	80	100

Parameters	Topology	Total component count = 16				
		Gain, Gv				
		8	10	16	20	
Duty cycle, D	Proposed converter	0.4	0.5	0.6	0.64	
	Ref^19^	0.5	0.6	0.75	0.8	

Parameters	Topology	Total component count = 16, Duty cycle = 0.5				
		Output voltage, Vo (kV)				
		1	2	5	10	
Input voltage, Vg, (V)	Proposed converter	100	200	500	1000	
	Ref^19^	125	200	625	1250	

It is noted that the suggested topology requires a low duty cycle, D, to achieve the required voltage gain compared to the converter chosen for comparison^19^, which ultimately reduces the conduction losses of the semiconductor losses and increases the efficiency of the converter. Next, the required input voltage to achieve the desired output voltage is compared by setting the duty cycle to 0.5 and fixing the total component count as 16. This study depicts in Fig. 11d that the derived topology requires lesser input voltage, Vg, compared to the topology in^19^ for various output voltages, Vo. Since the losses across the power semiconductor devices are more compared to the passive component’s losses, the HPSQB topology offers better efficiency compared to^19^. It is observed that to achieve the voltage gain of 10, the number of semiconductor devices required for^19^ is 10 whereas the proposed topology is 7.

Finally, the comparative study is extended by choosing similar topologies with diode-capacitor cell and extendable capability. Figure 11e illustrates the analysis carried out and it shows that the derived topology exhibits high gain compared to other topologies. The gain expression and the component breakup are depicted in Table 8. Furthermore, topologies in literature are extended to derive the high voltage pulses in bipolar mode^30^. This bipolar pulse generation is extended as a future scope with the suggested topology.

**Table 8:** Comparative study of the HPSQB converter with similar converters in literature.

Topology	Gain	Total component count				Suggested application
		Switch	Diode	Capacitor	Inductor	
Proposed converter	$\frac{M(2+D)}{(1-D{)}^{2}}$	1	4 + 2 M	4 + 2 M	3	Water treatment application
Ref^19^	$\frac{M+1}{1-D}$	1	1 + 2 M	1 + 2 M	1	Water treatment application
Ref^23^	$M{V}_{CM}$	1 + 2 M	1 + M	1 + M	0	Pulsed voltage application
Ref^28^	$\frac{1+(2M+3)D}{1-D}$	2 + M	6 + 5 M	1	4 + 2 M	Low input integrated application
Ref^29^	$\frac{2(1+MD)}{1-D}$	1	3(1 + M)	3	1 + M	Low voltage DC Source Integration
Ref^37^	$\frac{3-D}{(1-D{)}^{2}}$	1	6	6	3	Low voltage DC Source Integration
Ref^38^	$\frac{3-D}{(1-D{)}^{2}}$	2	4	4	2	Renewable power Source Integration

## Simulation results

**Figure 12:**
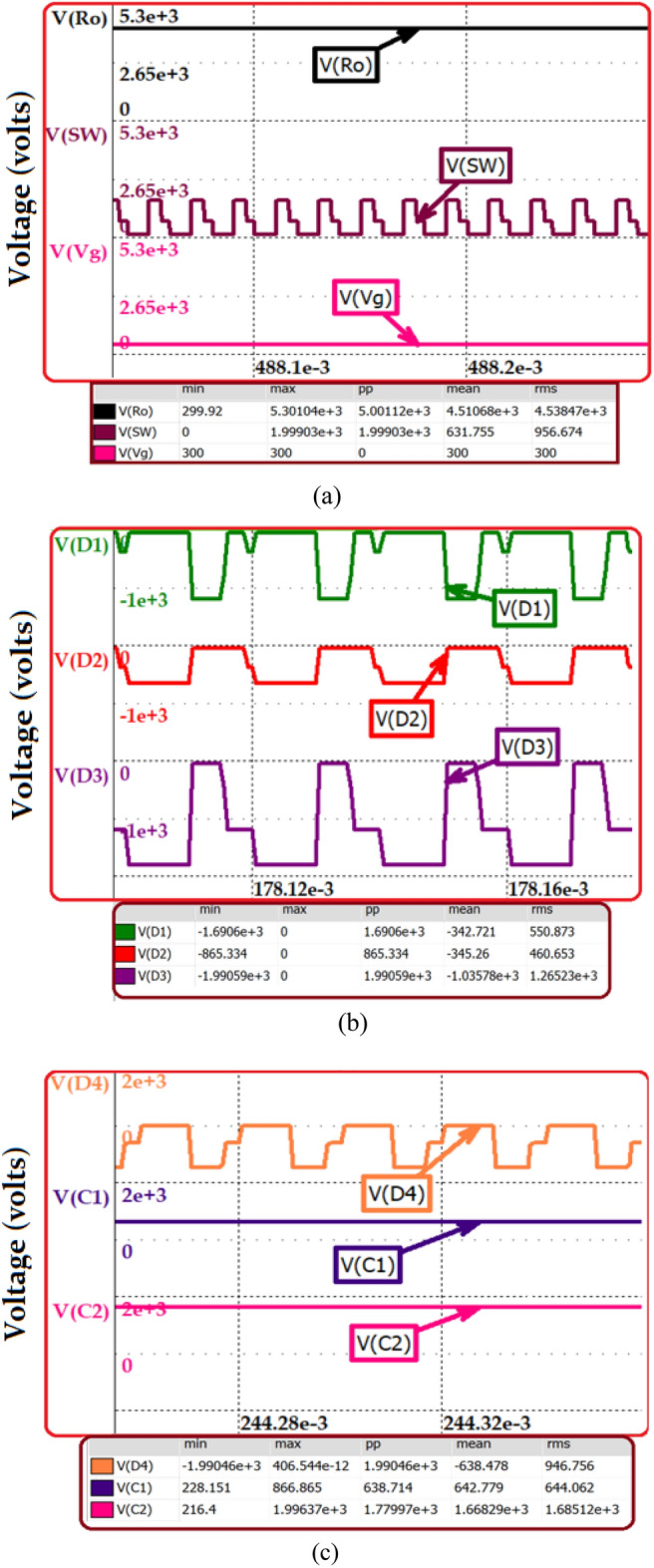

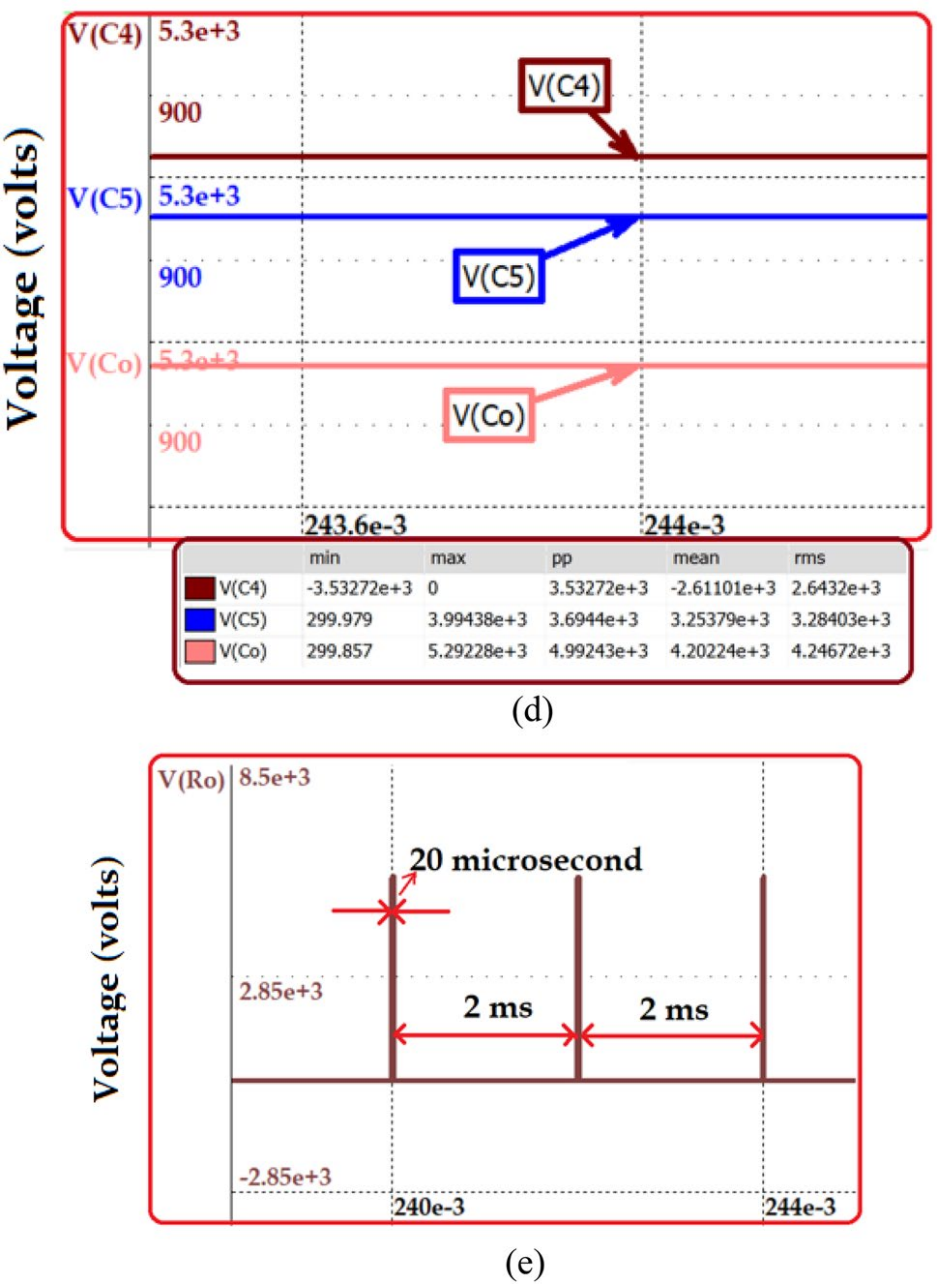
Simulation results (**a**) Output and switch voltage at steady state condition (**b**) Diode voltage (**c**) and (**d**) capacitor voltage (**e**) High pulse voltage.

The proposed topology is simulated with a 500 W power rating for generating a high voltage pulse of 5 kV. The specification of the derived topology is depicted in Table 9. The load resistance for the simulation study is obtained based on the dimension considered for analysis. The area of the plate and the distance between the plates are chosen as 1 cm and 1 cm, respectively. Considering the conductivity of water as 20 µs/cm, the equivalent resistance of the water sample is calculated as 50 kΩ. Figure 12a–e presents the simulation result obtained from the nL5 simulator.

**Table 9:** Simulation Parameters of the HPSQB topology.

Parameters	Value
Proposed converter	
Power	500 W
Input voltage	300 V
Duty ratio	0.61
Gain	17 times
Load resistance	50 kΩ
Switching frequency	50 kHz
Inductor L_1_, L_2_ and L_3_	5 mH, 20 mH, 100 mH
Capacitor C_1_–C_5_ and CO	10 µF, 1 µF
Output voltage	5 kV
Number of multiplier cell	One
Pulse width	200 µs
Repetitive rate of the pulses	500 pulses/sec
Load specification	
Distance between the electrode	1 cm
Area of the plate	1 cm^2^
Water conductivity	20 µs/cm
Load resistance (water sample)	50 kΩ
Switch to produce pulsed output	
Switching frequency	500 Hz
Duty cycle	1%

Figure 12a depicts the output and switch voltage before the pulse forming unit for 300 V input voltage. The voltage stress across the switch is validated with the analytical study, Vsw = Vg/(1–D)2 = 1.9 kV. Similarly, the output voltage for M = 1 and D = 0.5 is (2 + D)/(1–D)2 = 5.1 kV. The diode voltage is obtained and illustrated in Fig. 12b. The voltage across diode D2 is Vg(1–D) = 0.8 kV. The voltage across diodes D3 and D4 is Vg/(1–D)2 = 1.9 kV, which is presented in Fig. 12b,c. The capacitor voltage is presented in Fig. 12d. Finally, the output high pulse is obtained after incorporating pulse forming unit. The acquired high voltage pulse of 5 kV is depicted in Fig. 12e. The switching frequency of the switch added in pulse forming unit is 500 Hz and the duty cycle is 10%. This is validated in Fig. 12e with the pulse width of 20 µs and the time period of 2 ms. The diode voltage is obtained and illustrated in Fig. 12b. The voltage across diode D_2_ is Vg(1–D) = 0.8 kV. The Voltage across diodes D_3_ and D_4_ is Vg/(1–D)^2^ = 1.9 kV which are presented in Fig. 12b,c. The capacitor voltage is presented in Fig. 12d. Finally, the output high pulse is obtained after incorporating pulse forming unit. The acquired high voltage pulse of 5 kV is depicted in Fig. 12e. The switching frequency of the switch added in pulse forming unit is 500 Hz and the duty cycle is 10%. This is validated in Fig. 12e with the pulse width of 20 µs and the time period of 2 ms.

## Experimental results

**Figure 13:**
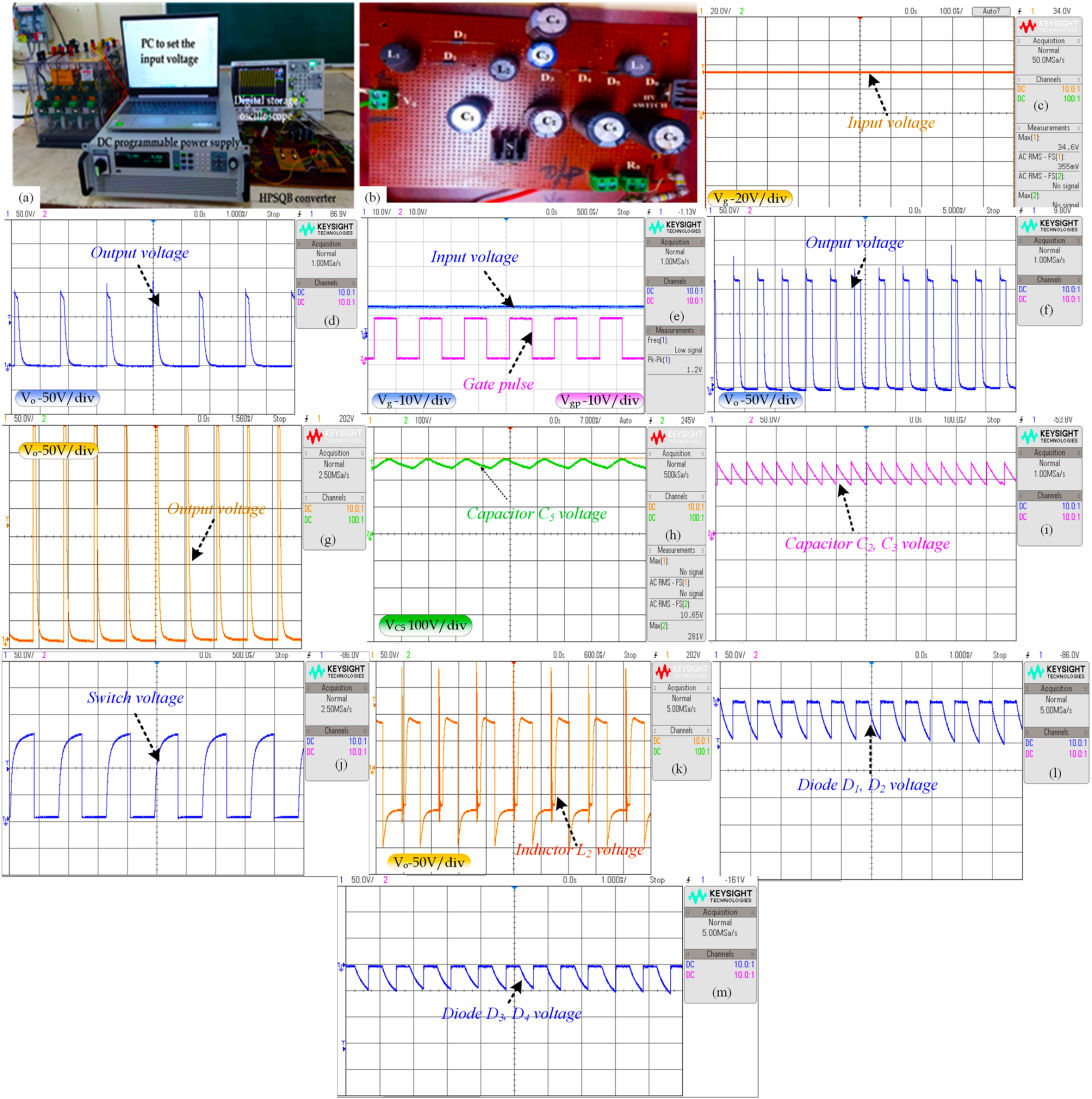
Test results (**a**) Photograph of the setup tested (**b**) Photograph of the converter (**c**) 20 V/div-Input voltage, V_g_ (**d**) 10 V/div-Input voltage, V_g_ and 10 V/div-Gate pulse, V_gate_ (**e**), (**f**) and (**g**) 50 V/div-Output voltage, Vo (**h**) 100 V/div-Capacitor voltage, V_C5_ (**i**) 50 V/div-Capacitor voltage, V_C2_ and V_C3_ (**j**) 50 V/div-Switch voltage, V_SW_ (**k**) 50 V/div- Inductor voltage, V_L2_ (**l**) 50 V/div-Diode voltage, V_D1_ and V_D2_ (**m**) 50 V/div-Diode voltage, V_D3_ and V_D4_.

The proposed converter is tested for 50 W power rating to validate the theoretical analysis carried out. The specification of the topology is presented in Table 10. The experimental study is scaled down to 50 W, 300 V for high pulse generation. The photograph of the setup tested is presented in Fig. 13a. The results obtained for the validation are presented in Fig. 13a–m. The input voltage varies from 12 to 36 V to obtain 120 V to 360 V results. The input voltage of 12 V, 24 V and 36 V is given to the converter, which is depicted in Fig. 13c,d. The duty cycle is kept constant for 0.5 and is observed in Fig. 13d. The corresponding high pulse generated from the inputs (12 V–36 V) is presented in Fig. 13e–g. The high voltage pulses of 120 V, 240 V and 360 V are presented in Fig. 13e–g, respectively. These output voltages are much suitable for treatment of microbes discussed in^21–23^. The capacitor C_5_ voltage (VC_5_) for the input 36 V is presented in Fig. 13h. It validates the steady-state analysis's theoretical result, which represents VC_5_ = 2Vg/(1–D)^2^. For 0.5 duty cycle and 36 V input, the capacitor voltage is 288 V which is justified in Fig. 13g. The capacitor C2 and C3 voltage (VC_2_ and VC_3_) is Vg/(1–D)^2^. This is validated for 24 V input voltage, and the result is presented in Fig. 13i. The voltage stress across the single switch in the suggested topology is observed for the input voltage of 36 V. The theoretical formula for the switch voltage stress is Vg/(1–D)^2^, which is validated in Fig. 13j. The inductor L2 voltage is presented in Fig. 13k to validate the volt-sec balance principle applied to the topology. Finally, the diode D1-D4 voltages are observed and presented in Fig. 13l–m.

**Table 10:** Specification of the laboratory HPSQB prototype.

Parameters	Value
Proposed converter	
Power	50 W
Input voltage	12 V to 36 V
Duty ratio	0.5
Gain	10 times
Load	1 kΩ
Switching frequency	50 kHz
Output voltage	120 V–360 V
Number of multiplier cell	One
Pulse width	200 µs
Repetitive rate of the pulses	500 pulses/sec
Switch to produce pulsed output	
Switching frequency	500 Hz
Duty cycle	1%

From the above sections, it is noted that the simulation results are obtained for 5 kV output voltage and hardware results are worked for 360 V output voltage. To validate the significance of these magnitude of voltage in water treatment, we planned to take water sample from our kitchen which is supplied from the municipality and decided to go for testing. The specification of the pulsed voltage considered for testing is given in Table 11. Raw water sample and the water samples subjected to 360 V and 5 kV in pulsed electric field chamber are tested in Indian analytical testing Lab, Bangalore. Finally, the obtained result from the lab before and after treatment is presented in Table 12. From the results, it is observed that all the microbes are absent after the treatment with 5 kV. With 360 V output voltage, the Coliform Bacteria is reduced from 10 cfu/gm to 2 cfu/gm whereas other microbes are completely absent.

**Table 11:** Specification of voltage considered for testing the sample.

Sl.no	Real time treatment conditions	Simulation and prototype obtained pulsed voltages	
		360 V (prototype output)	5 kV (simulation output)
1	Frequency of Pulses	500 Hz	500 Hz
2	Pulse width	168 μs	169 μs
3	Distance between the electrodes	9.87 mm	9.87 mm
4	Current	0.4 A	0.1 A
5	Treatment time	6 min	6 min
6	Type of electrode	Stainless steel	Stainless steel
7	Sample description	Raw water	Raw water
8	Place of collection	Kitchen	Kitchen
9	Treatment temperature	26 °C	29 °C
10	Treatment chamber	Static	Static

**Table 12:** Microbes identified in the sample and the test results.

Sl.no	Microbes identified	Sample-raw water (Municipality water collected from kitchen)		
		Before treatment	Treated under 360 V pulsed DC	Treated under 5000 V pulsed DC
1	Coliform bacteria	10 cfu/gm	02 cfu/gm	Absent
2	Staphylococcus auerus/gm	06 cfu/gm	Absent	Absent
3	Salmonella	02 cfu/gm	Absent	Absent
4	Escherichia coli	08 cfu/gm	Absent	Absent

## Conclusion

This paper validates the performance of high voltage pulse generating topology derived from quadratic and modified SEPIC converter with voltage multiplier cells for water treatment applications. A wide-ranging analysis of the operating principle and design of the HPSQB converter is conducted. The steady-state performance of the topology is mathematically proven with the test results. The derived topology generates a rectangular unipolar pulse with flexibility in the variation of pulse duration and amplitude. The proposed HPSQB converter has unique features such as (i) high gain is achieved with the lesser component count, (ii) it can be integrated into low voltage input supply even for domestic applications, (iii) more efficient and reliable, (iv) voltage stress across the power semiconductor devices is less. The comparative study indicates that the HPSQB converter requires a lesser component to achieve high voltage gain compared to similar topologies in the literature. The derived topology is extendable and flexible, where the desired gain of the converter is achieved by adjusting the number of multiplier cells, M. A scaled-down prototype of 50 W, 0.36 kV is tested to validate the analytical studies. Both experimental and simulation studies analyze the generation of the unipolar pulse from the pulse-forming unit. The obtained test results exhibit excellent accordance with theoretical and simulation analysis.

## Data Availability

The datasets used and/or analysed during the current study available from the corresponding author on reasonable request.

## Abbreviations

G_V_: Voltage gain
G_I_: Current gain
M: Number of Multiplier cell
CDVM: Capacitor–diode voltage multiplier
PEF: Pulsed Electric Field
PFN: Pulse Forming Network
D: Duty Cycle
Vg: Input voltage
Vo: Output voltage
V_Gate_: Gate pulse
V_C_: Capacitor voltage
V_L_: Inductor voltage
R_L_: Internal resistance of the inductor
R_O_: Load resistance
CCM: Continuous conduction mode
HPSQB: High pulse SEPIC–Quadratic Boost
